# Reduced chain length in myelin sphingolipids and poorer motor coordination in mice deficient in the fatty acid elongase *Elovl1*

**DOI:** 10.1096/fba.2019-00067

**Published:** 2019-11-22

**Authors:** Masashi Isokawa, Takayuki Sassa, Satoko Hattori, Tsuyoshi Miyakawa, Akio Kihara

**Affiliations:** ^1^ Laboratory of Biochemistry Faculty of Pharmaceutical Sciences Hokkaido University Sapporo Japan; ^2^ Division of Systems Medical Science Institute for Comprehensive Medical Science Fujita Health University Toyoake Japan

**Keywords:** galactosylceramide, lipid, spastic paraplegia, very‐long‐chain fatty acids

## Abstract

Very‐long‐chain fatty acids, with a chain length of >C20, are abundant in myelin sphingolipids. Recently, a de novo mutation in the *ELOVL1* gene, which encodes fatty acid elongase, was identified in patients with neurocutaneous disorders involving skin ichthyosis and multiple neurological abnormalities, including hypomyelination, spastic paraplegia, and high‐frequency deafness. However, the consequences of *ELOVL1* deficiency for lipid composition and detailed pathological changes in the brain remain unclear. Here, we analyzed *Elovl1* mutant mice as a model of human *ELOVL1* deficiency. The mice exhibited a decreased postnatal survival rate, and some died of startle epilepsy. The acyl chain length of sphingolipids such as galactosylceramides, sulfatides, sphingomyelins, and ceramides in the brains of these mice was markedly shortened. Moreover, the mice exhibited reduced levels of galactosylceramides, which are important for myelin formation and stability. Electron microscope analysis of the corpus callosum in *Elovl1* mutant mice revealed modest hypomyelination, especially in large‐diameter axons. Furthermore, behavioral testing of the mice revealed deficits such as poorer motor coordination and reduced acoustic startle response to high‐intensity stimulus. These findings provide clues to the molecular mechanism of the neurological symptoms of patients with the *ELOVL1* mutation.

**Abbreviations**
2‐OH: 2‐hydroxy
FA: fatty acid
GalCer: galactosylceramide
LC: liquid chromatography
MS/MS: tandem mass spectrometry
RT: reverse transcription
TLC: thin‐layer chromatography
VLC: very‐long‐chain

## 1 INTRODUCTION

Myelin, a multilayered stack of tightly apposed membranes around an axon, functions as an insulator and enables the saltatory propagation of action potentials for the rapid transmission of neural information.^1^ Myelin is essential for motor, sensory, and cognitive functions, as evidenced by the symptoms of many myelin‐related disorders, such as multiple sclerosis and leukodystrophy.^2^ Myelin is a highly lipid‐enriched structure, with its lipid content constituting 70‐80% of its dry weight.^3^ Sphingolipids are one of the most abundant lipids in myelin. They are composed of a hydrophobic backbone (ceramide) and a polar head group. Sphingolipids are classified into sphingophospholipids and glycosphingolipids according to differences in the polar head groups. Sphingomyelin, which has a phosphocholine moiety, is the primary mammalian sphingophospholipid. The most abundant sphingolipid in myelin is the glycosphingolipid galactosylceramide (GalCer), which has a galactose moiety.^4^ Myelin also contains sulfatide, which is sulfated at the C3 position of the galactose in GalCer, and which is 1/6 to 1/3 as abundant as GalCer.^3^ Mice deficient in the GalCer synthase *Ugt8a* lack both GalCer and sulfatide and exhibit splitting of the myelin lamellae, nerve‐conduction deficiency, and ataxia, demonstrating that these species are essential for myelin function and stability.^5^, ^6^

A remarkably large proportion of the fatty acid (FA) moieties of myelin sphingolipids are very‐long‐chain (VLC) FAs, with a carbon chain length of >C20.^7^, ^8^ VLCFAs are synthesized from long‐chain FAs (C11–C20) via the FA elongation cycle in the endoplasmic reticulum. Each FA elongation cycle is composed of four sequential reactions: condensation, reduction, dehydration, and reduction.^7^, ^8^ The first reaction is the condensation of acyl‐CoA with malonyl‐CoA, a donor of two carbon units, resulting in the synthesis of 3‐ketoacyl‐CoA. The subsequent three reactions convert 3‐ketoacyl‐CoA to acyl‐CoA in the following order: reduction to 3‐hydroxyacyl‐CoA, dehydration to *trans*‐2‐enoyl‐CoA, and finally reduction to acyl‐CoA. Thus, acyl‐CoA is elongated by two carbon units per cycle. The first condensation reaction is the rate‐limiting step of the cycle^9^, ^10^ and is catalyzed by the FA elongases called ELOVLs (elongation of very‐long‐chain FAs).^7^, ^8^ Mammals have seven ELOVL isozymes (ELOVL1 to ELOVL7); these exhibit characteristic but partially overlapping substrate specificities toward acyl‐CoAs, making it possible to synthesize acyl‐CoAs differing in chain length and the number of double bonds.^11^, ^12^ Of the seven isozymes, ELOVL1 is active toward saturated and monounsaturated C20‐ to C24‐CoAs in vitro.^12^, ^13^ The VLC acyl‐CoAs produced by ELOVL1 are mainly incorporated into sphingolipids throughout the body, although they are also used for the production of wax esters and cholesterol esters in the meibomian glands in the eyelids.^13^

To study the function of VLC lipids, we previously generated *Elovl1* knockout (*Elovl1* ^−/−^) mice.^14^ These mice exhibited an epidermal permeability barrier defect due to a decrease in acylceramide (ω‐*O*‐acylceramide).^14^ Acylceramide contains a C30–C36 VLCFA esterified with a linoleic acid at ω‐carbon and is essential for epidermal barrier formation.^15^, ^16^ We also found that *Elovl1* mutant mice showed a dry‐eye‐like phenotype due to the shortening of VLC wax and cholesterol esters secreted from the meibomian glands.^13^ These studies demonstrated the involvement of VLC lipids in barrier function on the body surface. However, the functions of VLC lipids in other tissues, such as the brain, are incompletely understood. Recently, a de novo mutation in the *ELOVL1* gene was identified in two patients with a neurocutaneous disorder.^17^, ^18^ These patients exhibited similar symptoms and carried an identical heterozygous mutation, which changed the Ser residue at 165 to Phe in the ELOVL1 protein.^17^, ^18^ Although the mutation abolished ELOVL1 enzymatic activity,^18^ it is unclear whether the disorder is caused by haploinsufficiency or an autosomal dominant mode of inheritance. The patients exhibited ichthyosis symptoms, which are associated with epidermal permeability‐barrier dysfunction, resemble the phenotype observed in *Elovl1* ^−/−^ mice and are likely explained by the reduction in VLC ceramides, especially acylceramides.^14^, ^17^, ^18^ The neurological abnormalities of these patients included hypomyelination in the brain, spastic paraplegia, and high‐frequency (>2000 Hz) hearing deficit.^17^, ^18^ One of the patients also exhibited photophobia. However, the link between the *ELOVL1* mutation and these neurological abnormalities remained unclear.

In this study, we analyzed the effects of *Elovl1* deficiency on the nervous system in mice. We found that the VLC acyl moieties in sphingolipids were significantly shortened in the brains of *Elovl1* ^−/−^ mice, and that their GalCer levels were decreased. The mutant mice exhibited hypomyelination of large‐diameter axons in the corpus callosum, mild impairment of their motor functions, and a reduced acoustic startle response to high‐intensity stimulus. The changes in sphingolipid chain length and/or composition in their brains may be associated with these abnormalities. Our results suggest that VLC sphingolipids in myelin are necessary for the development of fully functional brain circuitry.

## 2 MATERIALS AND METHODS

### 2.1 Mice and behavioral testing

In this study we analyzed *Elovl1* ^−/−^ *Tg*(*IVL‐Elovl1*) mice, in which the epidermal barrier defect and neonatal lethality was rescued by the transgenic expression of *Elovl1* in the epidermis.^13^ Mice were housed in a room kept at 23 ± 1°C with a 12 hours light/dark cycle (lights on at 07:00) with food and water available ad libitum. *Elovl1* ^+/−^ *Tg*(*IVL‐Elovl1*) mice were maintained by backcrossing with C57BL/6J mice. For behavioral testing, we generated male *Elovl1* ^+/+^ *Tg*(*IVL‐Elovl1*) and *Elovl1* ^−/−^ *Tg*(*IVL‐Elovl1*) mice by in vitro fertilization using sperm from a homozygous transgenic male *Elovl1* ^+/−^ *Tg*(*IVL‐Elovl1*) mouse and eggs from female *Elovl1* ^+/−^ mice. Mice used in this study were from at least the eighth generation of backcrossing. All mice were 8 weeks old at the start of the behavioral testing. Behavioral tests were carried out using protocols identical to previous reports,^19^ except for the acoustic startle response test, in which mice were given acoustic stimuli with intensities of 70, 80, 85, 90, 95, 100, 105, 110, and 120 dB. Behavioral testing was performed between 09:00 and 18:00. The animal experiments performed in this study were approved by the institutional animal care and use committees of Hokkaido University and Fujita Health University.

### 2.2 Quantitative real‐time reverse transcription (RT)‐PCR

Total RNAs were isolated from the whole brain using the NucleoSpin RNA kit (Macherey‐Nagel) according to the manufacturer's instructions. First‐strand cDNAs were synthesized using oligo (dT) primer and the PrimeScript II 1st strand cDNA Synthesis Kit (Takara Bio) according to the manufacturer's instructions. Quantitative real‐time PCR was performed using first‐strand cDNAs and KOD SYBR qPCR Mix (Toyobo) on a CFX96 Touch real‐time PCR detection system (Bio‐Rad). Forward (‐F) and reverse (‐R) primers for the respective genes were used (Table 1). The mRNA levels were normalized with respect to those of *GAPDH*. The reactions were conducted by incubating the samples at 98°C for 2 minutes, followed by 40 cycles of 98°C for 10 seconds, 60°C for 15 seconds, and 68°C for 1 minute.

**Table 1:** Primers used in this study

Primer	Sequence
*Elovl1*‐F	5′‐CATGCTTTCCAAGGTCATTGAGCTG‐3′
*Elovl1*‐R	5′‐TCTCAGTTGGCCTTGACCTTGGTGG‐3′
*Ugt8a*‐F	5′‐CACTGCCAGAAGATCTGCAGAGGTG‐3′
*Ugt8a*‐R	5′‐GAGCTTAGTGTTGTTTCCTAGGTTC‐3′
*Fa2h*‐F	5′‐GATGGGCCACCTCACAGACACTCCG‐3′
*Fa2h*‐R	5′‐CTCGATGAGGTCTGAGTGGAAGAGG‐3′
*Cers2*‐F	5′‐AAGTTCCGAGAAGCCAGCTGGAGAT‐3′
*Cers2*‐R	5′‐CAATGCTGAAGAGCAGGGACCAGTA‐3′
*Gapdh‐*F	5′‐GAACGGGAAGCTCACTGGCATGGCC‐3′
*Gapdh‐*R	5′‐TGTCATACCAGGAAATGAGCTTGAC‐3′

### 2.3 Lipid analyses

Chopped brain tissue was suspended in 40 μl of chloroform/methanol (1:2, v/v) per mg tissue and homogenized in a homogenizer. The homogenate was cleared by centrifugation at 1100 × *g* at room temperature for 5 minutes. Lipids were extracted from 900 µl of the cleared homogenate by sequential addition of 300 μl of chloroform and 540 μl of water with vigorous mixing. After centrifugation at 10 000 × *g* for 3 minutes, the organic phase was recovered and dried. The dried lipids were suspended in chloroform/methanol (2:1, v/v), and lipids corresponding to 1 mg of brain tissue were resolved by thin‐layer chromatography (TLC) on silica gel 60 high‐performance TLC plates (Merck), using 1‐butanol/acetic acid/water (3:1:1, v/v) as the solvent system, and stained with a cupric acetate/phosphoric acid solution.

Lipids corresponding to 5 µg of brain tissue [for GalCer, 2‐hydroxy (2‐OH) GalCer, sulfatide, and ceramide] or 0.5 µg of brain tissue (for sphingomyelin) were subjected to liquid chromatography (LC)–tandem mass spectrometry (MS/MS) analysis using an ultra‐performance LC–triple‐quadrupole mass spectrometry system (Xevo TQ‐S; Waters), as described previously.^20^ Lipids were resolved on a reverse‐phase column (ACQUITY UPLC CSH C18 column; 1.7 µm particle size, 2.1 × 100 mm; Waters), ionized by electrospray ionization in positive ion mode, and detected using multiple reaction monitoring by selecting the specific *m/z* at quadrupole mass filters Q1 and Q3 and using the appropriate collision energy for each GalCer, 2‐OH GalCer, sulfatide, sphingomyelin, and ceramide species (Tables 2, 3, 4). Data were analyzed and quantified using MassLynx software (Waters).

**Table 2:** Selected *m/z* values and collision energy for GalCer and 2‐OH GalCer species in LC‐MS/MS analyses

Lipid species	Precursor ions (Q1) [M−H_2_O+H]^+^	Product ion (Q3)	Collision energy (eV)
C12:0 GalCer*	626.6	264.3	40
C16:0 GalCer	682.6	264.3	40
C18:1 GalCer	708.6	264.3	40
C18:0 GalCer	710.6	264.3	40
C20:1 GalCer	736.6	264.3	40
C20:0 GalCer	738.6	264.3	40
C22:1 GalCer	764.6	264.3	40
C22:0 GalCer	766.6	264.3	40
C24:1 GalCer	792.7	264.3	40
C24:0 GalCer	794.7	264.3	40
C26:1 GalCer	820.7	264.3	40
C26:0 GalCer	822.7	264.3	40
2‐OH C16:0 GalCer	698.6	264.3	40
2‐OH C18:1 GalCer	724.6	264.3	40
2‐OH C18:0 GalCer	726.6	264.3	40
2‐OH C20:1 GalCer	752.6	264.3	40
2‐OH C20:0 GalCer	754.6	264.3	40
2‐OH C22:1 GalCer	780.6	264.3	40
2‐OH C22:0 GalCer	782.6	264.3	40
2‐OH C24:1 GalCer	808.7	264.3	40
2‐OH C24:0 GalCer	810.7	264.3	40
2‐OH C26:1 GalCer	836.7	264.3	40
2‐OH C26:0 GalCer	838.7	264.3	40

* External control

**Table 3:** Selected *m/z* values and collision energy for sulfatide and 2‐OH sulfatide species in LC‐MS/MS analyses

Lipid species	Precursor ions (Q1) [M−SO_3_−H_2_O+H]^+^	Product ion (Q3)	Collision energy (eV)
C16:0 sulfatide	682.6	264.3	40
C18:1 sulfatide	708.6	264.3	40
C18:0 sulfatide	710.6	264.3	40
C20:1 sulfatide	736.6	264.3	40
C20:0 sulfatide	738.6	264.3	40
C22:1 sulfatide	764.6	264.3	40
C22:0 sulfatide	766.6	264.3	40
C24:1 sulfatide	792.7	264.3	40
C24:0 sulfatide	794.7	264.3	40
C26:1 sulfatide	820.7	264.3	40
C26:0 sulfatide	822.7	264.3	40
2‐OH C16:0 sulfatide	698.6	264.3	40
2‐OH C18:1 sulfatide	724.6	264.3	40
2‐OH C18:0 sulfatide	726.6	264.3	40
2‐OH C20:1 sulfatide	752.6	264.3	40
2‐OH C20:0 sulfatide	754.6	264.3	40
2‐OH C22:1 sulfatide	780.6	264.3	40
2‐OH C22:0 sulfatide	782.6	264.3	40
2‐OH C24:1 sulfatide	808.7	264.3	40
2‐OH C24:0 sulfatide	810.7	264.3	40
2‐OH C26:1 sulfatide	836.7	264.3	40
2‐OH C26:0 sulfatide	838.7	264.3	40

**Table 4:** Selected *m/z* values and collision energy for ceramide and sphingomyelin species in LC‐MS/MS analyses

Lipid species	Precursor ions (Q1) [M−H_2_O+H]^+^	Product ion (Q3)	Collision energy (eV)
C16:0 ceramide	520.6	264.3	32
C18:1 ceramide	546.6	264.3	32
C18:0 ceramide	548.6	264.3	32
C20:1 ceramide	574.6	264.3	32
C20:0 ceramide	576.6	264.3	32
C22:1 ceramide	602.6	264.3	32
C22:0 ceramide	604.6	264.3	32
C24:1 ceramide	630.7	264.3	32
C24:0 ceramide	632.7	264.3	32
C25:0 ceramide*	646.7	264.3	32
C26:1 ceramide	658.7	264.3	32
C26:0 ceramide	660.7	264.3	32
C12:0 sphingomyelin*	647.6	181.4	60
C16:0 sphingomyelin	703.6	181.4	60
C18:1 sphingomyelin	729.6	181.4	60
C18:0 sphingomyelin	731.6	181.4	60
C20:1 sphingomyelin	757.6	181.4	60
C20:0 sphingomyelin	759.6	181.4	60
C22:1 sphingomyelin	785.6	181.4	60
C22:0 sphingomyelin	787.6	181.4	60
C24:1 sphingomyelin	813.7	181.4	60
C24:0 sphingomyelin	815.7	181.4	60
C26:1 sphingomyelin	841.7	181.4	60
C26:0 sphingomyelin	843.7	181.4	60

* External control

### 2.4 Hematoxylin and eosin staining

Eyes of 16 week‐old mice were fixed with SUPER FIX (Kurabo) at 4°C for >24 hours. They were then processed for dehydration, embedding in paraffin, preparation of 4 µm sections, deparaffinization, rehydration, and staining with hematoxylin and eosin using an automated staining system (Tissue Tek DRS 2000, Sakura Finetek) as described previously.^19^ Bright‐field images were captured using a Leica DM5000B microscope equipped with a DFC295 digital color camera (Leica Microsystems).

### 2.5 Transmission electron microscopy

Brains isolated from 13‐month‐old mice were dissected and the parasagittal blocks (3 × 3 × 1 mm) containing the corpus callosum were fixed with 2% paraformaldehyde, 2% glutaraldehyde, 2% osmium tetroxide, and 2.5% sucrose in 0.1 M sodium cacodylate buffer (pH 7.4) at 4°C for 3 hours. Fixed samples were dehydrated in graded ethanol, infiltrated with propylene oxide, and embedded in Quetol‐812 resin (Nisshin EM). The samples were ultrathin‐sectioned at 70 nm on a microtome (Leica Ultracut UCT; Leica Microsystems), mounted on copper grids, and stained sequentially with 2% uranyl acetate and lead staining solution (Merck). They were observed with a transmission electron microscope (JEM‐1400Plus; JEOL) equipped with a digital camera (EM‐14830RUBY2; JEOL).

### 2.6 Quantification and statistical analyses

Data are presented as means ± SD or means ± SEM. Data analyses were performed using StatView 5.0 (SAS Institute, Cary, NC, USA) and JMP13.1 (SAS Institute). Between‐group differences were evaluated using Student's *t* test, one‐way ANOVA, or two‐way repeated measures ANOVA. *P‐*values of <.05 were considered significant.

## 3 RESULTS

### 3.1 Decreased acyl chain lengths and amounts of brain sphingolipids in *Elovl1*‐deficient mice

**Figure 1:**
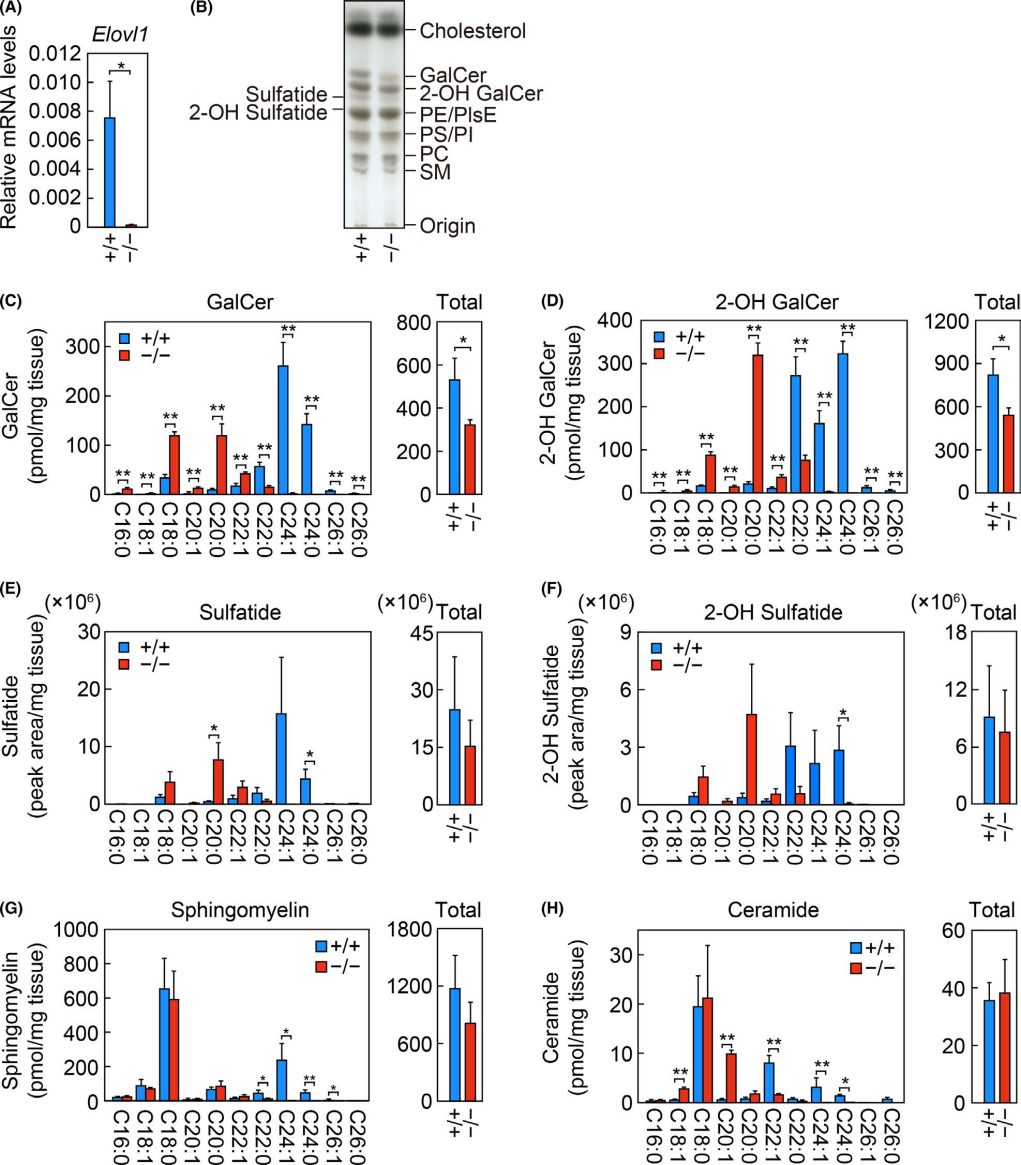
Decreased acyl chain lengths and levels of brain sphingolipids in *Elovl1* ^−/−^ *Tg*(*IVL‐Elovl1*) mice. A) Total RNA prepared from the brains of two‐ to three‐month‐old control [*Elovl1* ^+/+^ *Tg*(*IVL‐Elovl1*)] (n = 3) and *Elovl1* ^−/−^ *Tg*(*IVL‐Elovl1*) (n = 3) mice were subjected to RT, followed by SYBR Green‐based quantitative real‐time PCR using specific primers that detect both transgene‐derived and endogenous *Elovl1* mRNAs. Values are the amount of total *Elovl1* mRNA relative to that of *Gapdh* and represent the means ± SD. B) Lipids were extracted from the brains of control and *Elovl1* ^−/−^ *Tg*(*IVL‐Elovl1*) mice, separated by TLC, and stained with a cupric acetate/phosphoric acid solution. PC, phosphatidylcholine; PI, phosphatidylinositol; PE, phosphatidylethanolamine; PlsE, plasmanyl/plasmenyl ethanolamine; PS, phosphatidylserine. C–H) Lipids were extracted from the brains of control (n = 3) and *Elovl1* ^−/−^ *Tg*(*IVL‐Elovl1*) (n = 3) mice, and GalCer (C), 2‐OH GalCer (D), sulfatide (E), 2‐OH sulfatide (F), sphingomyelin (G), and ceramide (H) species were analyzed by LC‐MS/MS. The left‐hand graphs in (C–H) show the amount of each species containing an FA moiety of the indicated chain length and degree of saturation. The right‐hand graphs in (C–H) show the total amounts of all species. Values represent the means ± SD. Asterisks indicate significant differences based on Student's *t* test (*, *P* < .05; **, *P* < .01). +/+, *Elovl1* ^+/+^ *Tg*(*IVL‐Elovl1*) mice; −/−, *Elovl1* ^−/−^ *Tg*(*IVL‐Elovl1*) mice

The *Elovl1* ^−/−^ *Tg*(*IVL‐Elovl1*) mice, which are deficient in *Elovl1* throughout the body except the epidermis, harbor the transgene *Tg*(*IVL‐Elovl1*) expressing 3×FLAG‐tagged *ELOVL1* under the control of the epidermis‐specific human *IVL* (involucrin) promoter. We have previously shown, by immunoblot analysis, that 3×FLAG‐ELOVL1 protein is expressed in the epidermis but not in the brain.^13^ To confirm the absence of transgene expression in the brain of *Elovl1* ^−/−^ *Tg*(*IVL‐Elovl1*) mice at the transcriptional level, we conducted quantitative real‐time RT‐PCR analysis using primers that detect both endogenous and transgene‐derived *Elovl1* mRNAs. The levels of *Elovl1* mRNA in *Elovl1* ^−/−^ *Tg*(*IVL‐Elovl1*) mice were close to the detection limit and significantly lower than in control [*Elovl1* ^+/+^ *Tg*(*IVL‐Elovl1*)] mice (Figure 1A). Thus, *Elovl1* ^−/−^ *Tg*(*IVL‐Elovl1*) mice are useful for studying the function of *Elovl1* in the brain.

To examine the effect of diminished *Elovl1* expression on lipid composition, we performed a TLC analysis of total lipids in the brain. The levels of both the non‐hydroxy and the 2‐OH forms of the glycosphingolipid GalCer were apparently decreased in *Elovl1* ^−/−^ *Tg*(*IVL‐Elovl1*) mice, whereas those of other lipids were nearly unchanged (Figure 1B). Next, we performed LC‐MS/MS analyses to investigate the effect of *Elovl1* deficiency on the chain length of sphingolipids. GalCer and 2‐OH GalCer mostly consisted of C22:0, C24:1, and C24:0 VLC species in control mice (Figure 1C, D). In *Elovl1* ^−/−^ *Tg*(*IVL‐Elovl1*) mice, the levels of these VLC GalCer and 2‐OH GalCer species were substantially decreased, with ≥C24 species being almost undetected, whereas C22:1 and ≤C20 species were significantly increased. Consistent with the results of the TLC analysis, the total levels of GalCer and 2‐OH GalCer were reduced by 39% and 34%, respectively, in *Elovl1* ^−/−^ *Tg*(*IVL‐Elovl1*) mice relative to control mice. We also quantified sulfatides and 2‐OH sulfatides (sulfated forms of GalCer and 2‐OH GalCer, respectively). Their FA composition resembled that of their respective non‐sulfated forms: they mostly consisted of C22:0, C24:1, and C24:0 VLC species in control mice, but were shortened to C22:1, C20:0, and C18:0 species in *Elovl1* ^−/−^ *Tg*(*IVL‐Elovl1*) mice (Figure 1E, F). The total levels of sulfatides and 2‐OH sulfatides did not differ significantly between these two groups of mice, although there was a slight reduction tendency in the sulfatides in the latter group. We then quantified sphingomyelin and ceramide species. The levels of C22:0 and ≥C24 VLC sphingomyelin species were decreased in *Elovl1* ^−/−^ *Tg*(*IVL‐Elovl1*) mice compared with control mice, while the levels of other species were almost unchanged (Figure 1G). Among ceramides, the levels of ≥C22 VLC species were decreased, whereas C18:1 and C20:1 species were increased (Figure 1H). The total levels of sphingomyelin and ceramide were comparable between control and *Elovl1* ^−/−^ *Tg*(*IVL‐Elovl1*) mice. These results indicate that *Elovl1* deficiency in the brain caused shortening of the acyl chain lengths of VLC sphingolipids to ≤C20 and decreases in the total amounts of GalCer and 2‐OH GalCer.

### 3.2 Decreased expression of the GalCer synthase *Ugt8a* in the brains of *Elovl1‐*deficient mice

**Figure 2:**
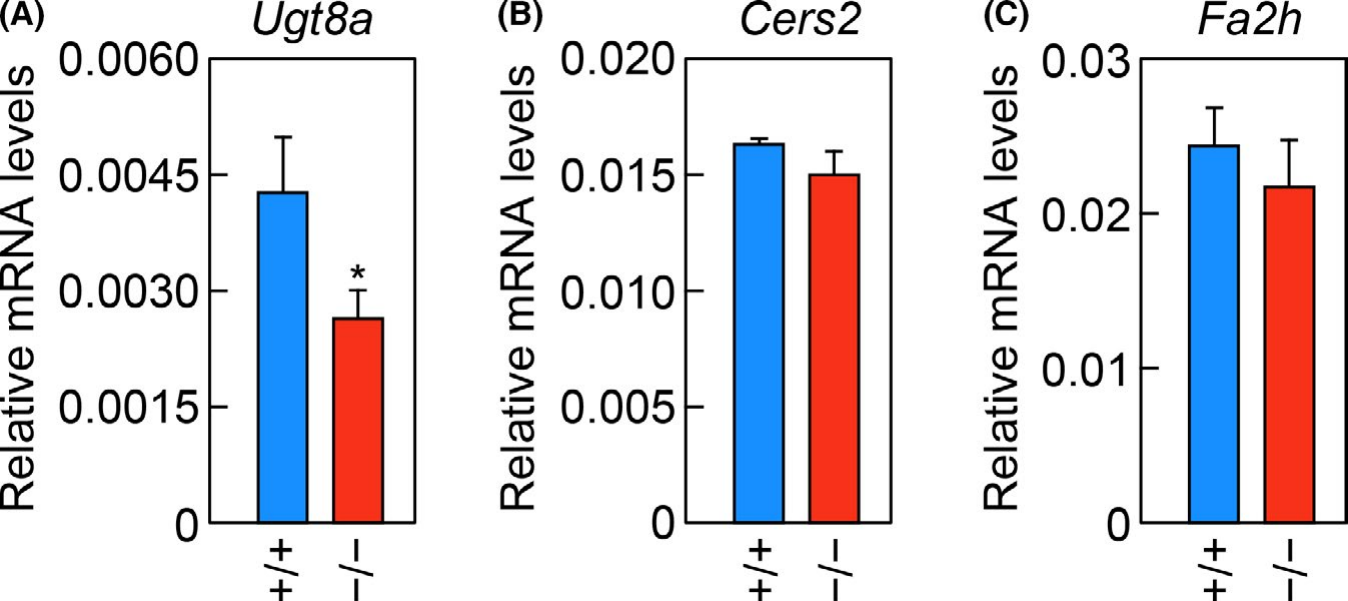
Decreased levels of *Ugt8a* mRNA in the brains of *Elovl1* ^−/−^ *Tg*(*IVL‐Elovl1*) mice. A–C) Total RNA prepared from the brains of two‐ to three‐month‐old male/female *Elovl1* ^+/+^ *Tg*(*IVL‐Elovl1*) (n = 3) and *Elovl1* ^−/−^ *Tg*(*IVL‐Elovl1*) (n = 3) mice were subjected to RT, followed by SYBR Green‐based quantitative real‐time PCR using specific primers that detect *Ugt8a* (A), *Cers2* (B), and *Fa2h* (C) mRNAs. Values are the amount of each mRNA relative to that of *Gapdh*, and represent the means ± SD. The asterisk indicates a significant difference based on Student's *t* test (*, *P* < .05). +/+, *Elovl1* ^+/+^ *Tg*(*IVL‐Elovl1*) mice; −/−, *Elovl1* ^−/−^ *Tg*(*IVL‐Elovl1*) mice

To elucidate the mechanism of the reduction of GalCer and 2‐OH GalCer levels, the mRNA levels of genes involved in their synthesis in the brain were examined by quantitative real‐time RT‐PCR. We examined the mRNA levels of *Ugt8a*, *Cers2*, and *Fa2h*. *Ugt8a* encodes the GalCer synthase UDP galactosyltransferase 8.^6^, ^21^ *Cers2* encodes one of the six mammalian ceramide synthases and is involved in the synthesis of VLC ceramides.^22^, ^23^ *Fa2h* encodes FA 2‐hydroxylase, which produces 2‐OH FA,^24^, ^25^ and mutations in the human *FA2H* gene cause hereditary spastic paraplegia (SPG35).^26^ These genes are highly expressed in oligodendrocytes in the brain.^5^, ^25^, ^27^ There was a significant reduction in the mRNA levels of *Ugt8a* in *Elovl1* ^−/−^ *Tg*(*IVL‐Elovl1*) mice of 38% relative to control mice, while the levels of *Cers2* and *Fa2h* were unchanged (Figure 2A–C). *Ugt8a* ^+/−^ mice had reduced levels of GalCer and 2‐OH GalCer compared with *Ugt8a* ^+/+^ mice,^6^ indicating that the mRNA levels of *Ugt8a* are correlated with the levels of GalCer and 2‐OH GalCer. Therefore, reductions in GalCer and 2‐OH GalCer levels in *Elovl1* ^−/−^ *Tg*(*IVL‐Elovl1*) mice may be partially attributable to the reduced mRNA levels of *Ugt8a*.

### 3.3 Thinning of myelin sheath in large‐diameter axons in *Elovl1*‐deficient mice

**Figure 3:**
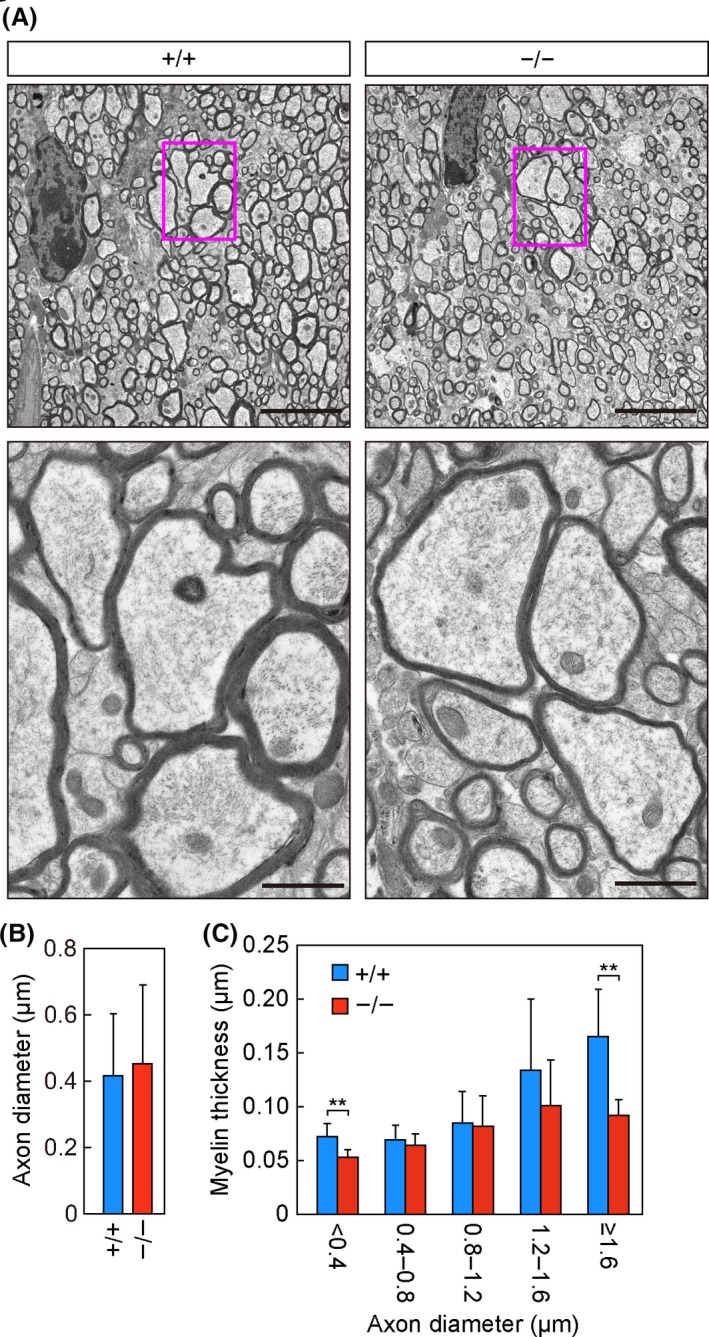
Thinning of the myelin sheath in *Elovl1* ^−/−^ *Tg*(*IVL‐Elovl1*) mice. A–C) Transmission electron microscopy analysis of the corpus callosum in *Elovl1* ^+/+^ *Tg*(*IVL‐Elovl1*) and *Elovl1* ^−/−^ *Tg*(*IVL‐Elovl1*) mice at 13 months of age. A) Representative images of the sagittal sections of the corpus callosum at low (5 µm, upper) or high (1 µm, lower) magnification. Red rectangles in upper panels indicate the area enlarged in lower panels. B) Average diameters ± SD of myelinated axons: n = 96 [*Elovl1* ^+/+^ *Tg*(*IVL‐Elovl1*)] and n = 73 [*Elovl1* ^−/−^ *Tg*(*IVL‐Elovl1*)] total axons counted in 13 different fields of view. C) Myelin thickness of axons counted in (B) was measured and presented according to axon diameter. Values represent the means ± SD. Asterisks indicate significant differences based on Student's *t* test (**, *P* < .01). +/+, *Elovl1* ^+/+^ *Tg*(*IVL‐Elovl1*) mice; −/−, *Elovl1* ^−/−^ *Tg*(*IVL‐Elovl1*) mice

We performed transmission electron microscopy analysis of the corpus callosum to examine whether the changes in brain sphingolipids described above were associated with white matter abnormalities like the hypomyelination seen in patients with the *ELOVL1* mutation.^17^, ^18^ There was no apparent change in the density of axons (Figure 3A), and the average axon diameter in control and *Elovl1* ^−/−^ *Tg*(*IVL‐Elovl1*) mice was similar (Figure 3B). The thickness of myelin increased in proportion to axon diameter in control mice (Figure 3C); however, in *Elovl1* ^−/−^ *Tg*(*IVL‐Elovl1*) mice such proportionality was less obvious, and the myelin was significantly thinner in axons with diameters of <0.4 µm or ≥1.6 µm, the latter being more severely affected (Figure 3A, C).

### 3.4 Reduced postnatal viability, body weight, motor function, and acoustic startle response in *Elovl1‐*deficient mice

*Elovl1* ^−/−^ *Tg*(*IVL‐Elovl1*) mice exhibited myelin thinning, which is related to the hypomyelination seen in patients with the *ELOVL1* mutation; however, these mice did not exhibit any noticeable signs of movement abnormalities related to spastic paraplegia. Therefore, we conducted a battery of behavioral tests to assess their motor functions in detail. To obtain a sufficient number of age‐matched *Elovl1* ^+/+^ *Tg*(*IVL‐Elovl1*) and *Elovl1* ^−/−^ *Tg*(*IVL‐Elovl1*) mice for these behavioral analyses, we performed in vitro fertilization using sperm from a homozygous transgenic male *Elovl1* ^+/−^ *Tg*(*IVL‐Elovl1*) mouse and eggs from female *Elovl1* ^+/−^ mice, and we obtained 265 newborn mice. Three weeks later, when genotyping was performed, the number of mice had decreased to 229 (Table 5). Of these, the genotype was successfully determined for 224. There were 22 *Elovl1* ^−/−^ *Tg*(*IVL‐Elovl1*) mice, which was one‐third of the expected number of 66 (= 265/4) based on the Mendelian ratio. There were 66 and 136 *Elovl1* ^+/+^ *Tg*(*IVL‐Elovl1*) and *Elovl1* ^+/−^ *Tg*(*IVL‐Elovl1*) mice, respectively. These numbers were close to the expected totals of 66 and 133, indicating that most of the mice lost during the three weeks after birth were *Elovl1* ^−/−^ *Tg*(*IVL‐Elovl1*) individuals. Three more of these mice were lost by the age of eight weeks, and two of them died from startle epilepsy. Although the exact mechanism of this lethality during the juvenile period is unclear, these results suggest that *Elovl1* plays an important role in survival.

**Table 5:** Numbers of *Elovl1^+/+^ Tg(IVL‐Elovl1), Elovl1^+/−^ Tg(IVL‐Elovl1),* and *Elovl1^−/−^ Tg(IVL‐Elovl1)* mice at birth and three weeks after birth

at birth						265
at three weeks	genotype	*Elovl1^+/+^ Tg(IVL‐Elovl1)*	*Elovl1^+/−^ Tg(IVL‐Elovl1)*	*Elovl1^−/−^ Tg(IVL‐Elovl1)*	unknown	total
	male	41	61	12	2	116
	female	25	75	10	3	113
	total	66	136	22	5	229

**Figure 4:**
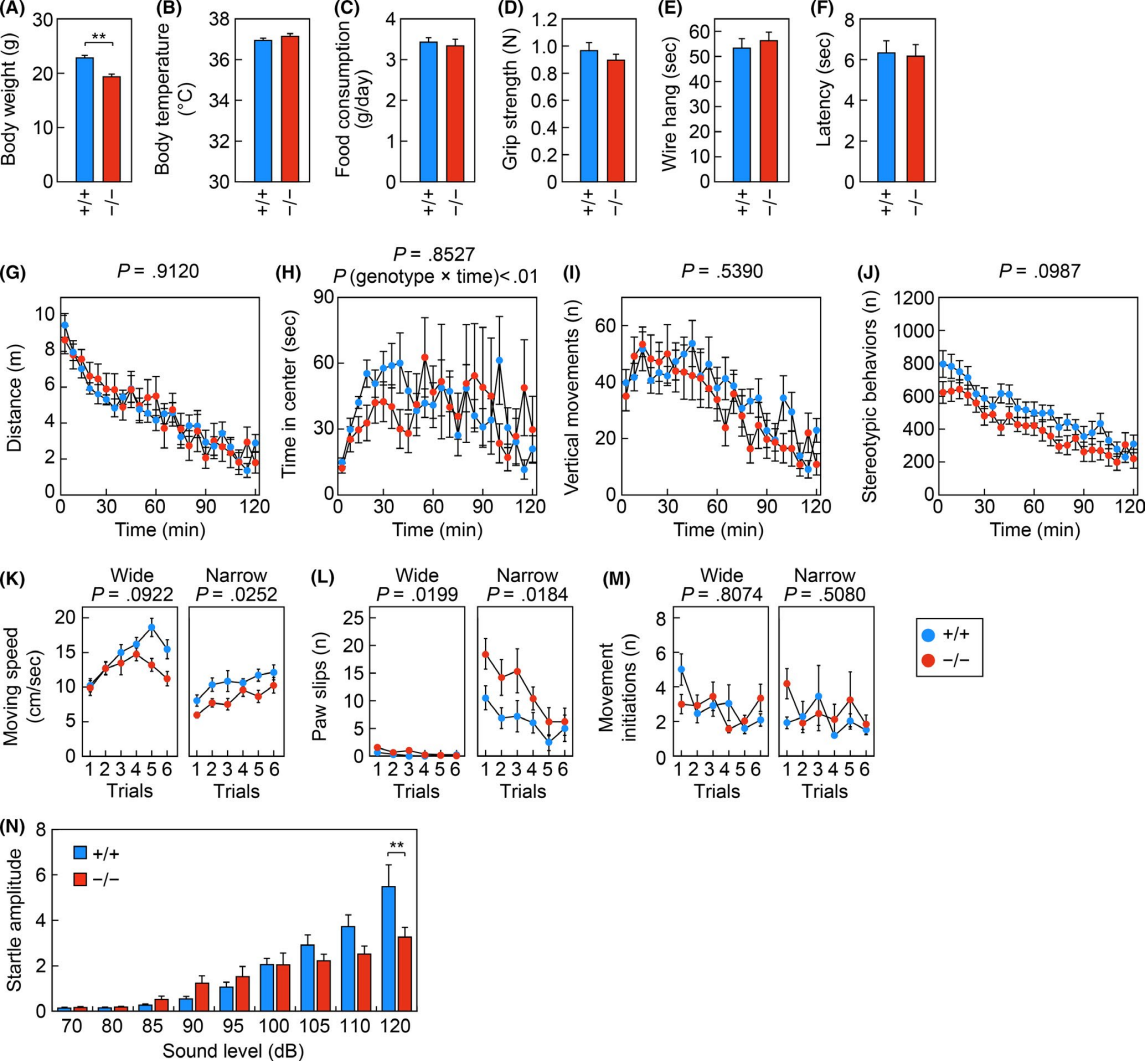
Reduced body weight, impaired motor function, and reduced acoustic startle response in *Elovl1* ^−/−^ *Tg*(*IVL‐Elovl1*) mice. *Elovl1* ^+/+^ *Tg*(*IVL‐Elovl1*) and *Elovl1* ^−/−^ *Tg*(*IVL‐Elovl1*) mice were assessed using the following metrics and tests: body weight (A), body temperature (B), food consumption (C), grip strength (D), wire hang latency (E), hot plate latency (F), the open‐field test (G–J), the balance beam test (K–M), and the acoustic startle response test (N). Open‐field test: distance traveled (G), time spent in the central area (H), number (*n*) of vertical movements (I), and number (*n*) of stereotypic behaviors (J*)*. Balance beam test: travel speed (K), number (*n*) of paw slips (L), and number (*n*) of movement initiations (M) on a wide (left) or narrow (right) beam. Error bars represent SEM. Asterisks indicate significant differences based on one‐way ANOVA (**, *P* < .01; A–F, N). *P*‐values correspond to the genotype effect in two‐way repeated measures ANOVA (G–M). *P* (genotype × time) in (H) corresponds to the *P*‐value for the interaction of genotype and time in a two‐way repeated measures ANOVA. Number of mice analyzed: n = 12 for both genotypes (A–E), n = 12 for *Elovl1* ^+/+^ *Tg*(*IVL‐Elovl1*) and n = 11 for *Elovl1* ^−/−^ *Tg*(*IVL‐Elovl1*) mice (F–M), n = 12 for *Elovl1* ^+/+^ *Tg*(*IVL‐Elovl1*) and n = 10 for *Elovl1* ^−/−^ *Tg*(*IVL‐Elovl1*) mice (N). +/+, *Elovl1* ^+/+^ *Tg*(*IVL‐Elovl1*) mice; −/−, *Elovl1* ^−/−^ *Tg*(*IVL‐Elovl1*) mice

The behavioral tests were conducted on male control [*Elovl1* ^+/+^ *Tg*(*IVL‐Elovl1*)] and *Elovl1* ^−/−^ *Tg*(*IVL‐Elovl1*) mice from when they were eight weeks old until they were 16 weeks old, well before the development of corneal opacity, as observed previously.^13^ The body weight of *Elovl1* ^−/−^ *Tg*(*IVL‐Elovl1*) mice was about 15% smaller than that of control mice (Figure 4A). There were no significant differences between the two groups of mice in terms of body temperature, food consumption, grip strength, muscular endurance in the wire hang test, or pain sensitivity in the hot plate test (Figure 4B–F). In the open‐field test, which measures locomotor activity and emotionality in a novel environment, *Elovl1* ^−/−^ *Tg*(*IVL‐Elovl1*) mice exhibited comparable responses with control mice in terms of total travel distance, time spent in the central area, number of vertical movements, and number of stereotypic behaviors (Figure 4G–J). However, the time course of time spent in the central area was significantly different between the two groups of mice, with *Elovl1* ^−/−^ *Tg*(*IVL‐Elovl1*) mice showing a tendency to spend less time in this area during the first 45 minutes of the test (Figure 4H). These results suggest that the basic motor functions are normal in *Elovl1* ^−/−^ *Tg*(*IVL‐Elovl1*) mice, but that they exhibit a behavioral preference for corners, which could be related to the photophobia seen in one patient with the *ELOVL1* mutation.

The mice were then subjected to the balance beam test. This test is useful for detecting mild deficits in motor balance and coordination. Each mouse was placed on one end of a wide or narrow beam (2.5 or 0.8 cm in diameter, respectively), and their movement toward a dark box at the opposite end was observed. On the wide beam, there were no significant differences between control and *Elovl1* ^−/−^ *Tg*(*IVL‐Elovl1*) mice; however, on the narrow beam, *Elovl1* ^−/−^ *Tg*(*IVL‐Elovl1*) mice moved significantly more slowly and exhibited more paw slips (Figure 4K, L). No significant difference was observed between the two groups of mice in the number of movement initiations on the beam (Figure 4M). These results suggest that *Elovl1* ^−/−^ *Tg*(*IVL‐Elovl1*) mice exhibit mild deficits in motor function.

The mice were next subjected to the acoustic startle response test. The mice were given a pulse (50 milliseconds) of white noise with various sound intensities, and their startle responses to the stimuli were measured. Control mice increased the amplitude of their startle response in proportion to the sound intensity (Figure 4N); however, the startle amplitude of *Elovl1* ^−/−^ *Tg*(*IVL‐Elovl1*) mice was less responsive to increases in sound intensity, at 120 dB it was significantly smaller than that of control mice (Figure 4N). These results suggest that *Elovl1* ^−/−^ *Tg*(*IVL‐Elovl1*) mice may exhibit defective audition.

### 3.5 Reduced activity in the light–dark transition test in *Elovl1*‐deficient mice

**Figure 5:**
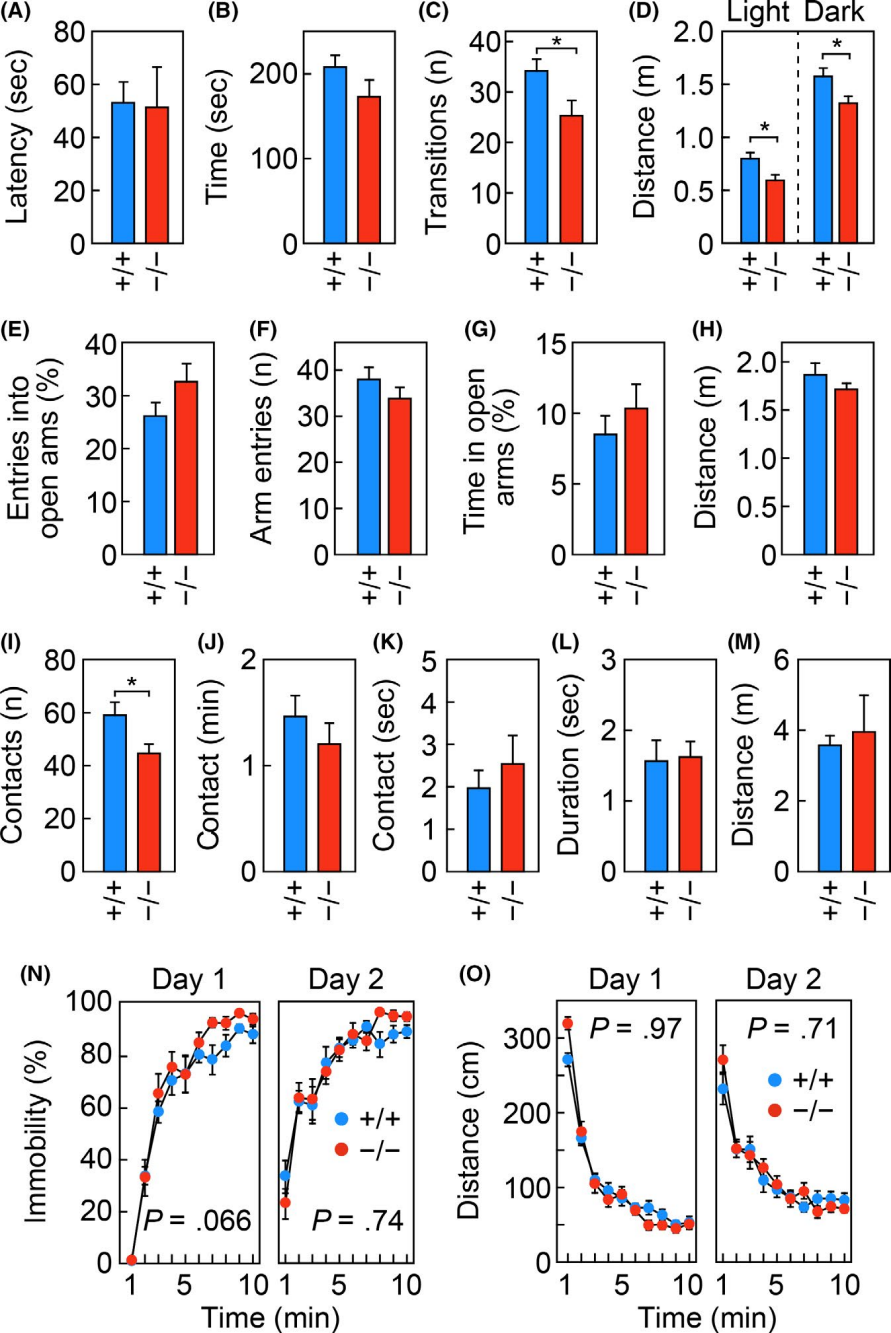
Reduced activity in the light–dark transition test in *Elovl1* ^−/−^ *Tg*(*IVL‐Elovl1*) mice. *Elovl1* ^+/+^ *Tg*(*IVL‐Elovl1*) (n = 12) and *Elovl1* ^−/−^ *Tg*(*IVL‐Elovl1*) (n = 11) mice were subjected to the light–dark transition test (A–D), the elevated plus‐maze test (E–H), the social‐interaction test in a novel environment (I–M), and the Porsolt forced‐swim test (N, O). Light–dark transition test: latency in entering the light chamber (A), time spent in the light chamber (B), number (*n*) of light–dark transitions (C), and distance traveled in the light and dark chambers (D). Elevated plus‐maze test: percentage of entries into open arms (E), number (*n*) of arm entries (F), percentage of time spent in open arms (G), and total distance traveled (H). Social‐interaction test in a novel environment: number (*n*) of contacts (I), total duration of contacts (J), total duration of active contacts (K), mean duration of each contact (L), and total distance traveled (M). Porsolt forced‐swim test: percentage of time spent immobile (N) and distance traveled (O). Error bars represent SEM. Asterisks indicate significant differences based on one‐way ANOVA (*, *P* < .05) (A–M). *P‐*values correspond to the genotype effect in a two‐way repeated measures ANOVA (N, O). +/+, *Elovl1* ^+/+^ *Tg*(*IVL‐Elovl1*) mice; −/−, *Elovl1* ^−/−^ *Tg*(*IVL‐Elovl1*) mice

In addition to spastic paraplegia of the lower limbs, hypomyelination in the brain, and a hearing deficit at high frequencies, patients with the *ELOVL1* mutation exhibit thinning of the nerve fiber layer in the retina, decreased visual acuity, and astigmatism; one patient also exhibited photophobia.^17^, ^18^ Therefore, we performed other tests that evaluate neurological functions not limited to motor and auditory functions. The mice were subjected to the light–dark transition test to examine their response to light. In this test, each mouse was first placed in the dark chamber, then allowed to move freely between the light and dark chambers. *Elovl1* ^−/−^ *Tg*(*IVL‐Elovl1*) mice exhibited normal anxiety behavior in response to light, and there were no significant differences between control and *Elovl1* ^−/−^ *Tg*(*IVL‐Elovl1*) mice in terms of latency before entering the light chamber for the first time or the amount of time spent in the light chamber (Figure 5A, 5B). However, *Elovl1* ^−/−^ *Tg*(*IVL‐Elovl1*) mice were less active in the test regardless of the luminance; they exhibited fewer transitions between the two chambers and reduced travel distance in both chambers (Figure 5C, 5D). As another test of anxiety‐like behavior, we performed the elevated plus‐maze test. *Elovl1* ^−/−^ *Tg*(*IVL‐Elovl1*) mice did not display any differences from control mice in the number of entries into open arms, the total number of entries into open or closed arm, the amount of time spent in open arms, or the travel distance (Figure 5E–H).

In the test of social interaction in a novel environment, two mice with the same genotype were placed together in the same box, and their behaviors were monitored. *Elovl1* ^−/−^ *Tg*(*IVL‐Elovl1*) mice exhibited a slight but significant decrease in the number of contacts compared with control mice (Figure 5I), whereas there were no significant differences between the two groups of mice in the total duration of contact, duration of active contact, mean duration per contact, or travel distance (Figure 5J–M). Finally, the mice were subjected to the Porsolt forced‐swim test, in which they were placed in a cylinder filled with water. *Elovl1* ^−/−^ *Tg*(*IVL‐Elovl1*) mice exhibited normal depression‐like responses, including the percentage of time spent immobile and travel distance (Figure 5N, O). These results suggest that *Elovl1* ^−/−^ *Tg*(*IVL‐Elovl1*) mice may dislike changes in luminance more than control mice, and that they have a subtle defect in sociality.

### 3.6 Normal retinal and corneal architecture in *Elovl1*‐deficient mice

**Figure 6:**
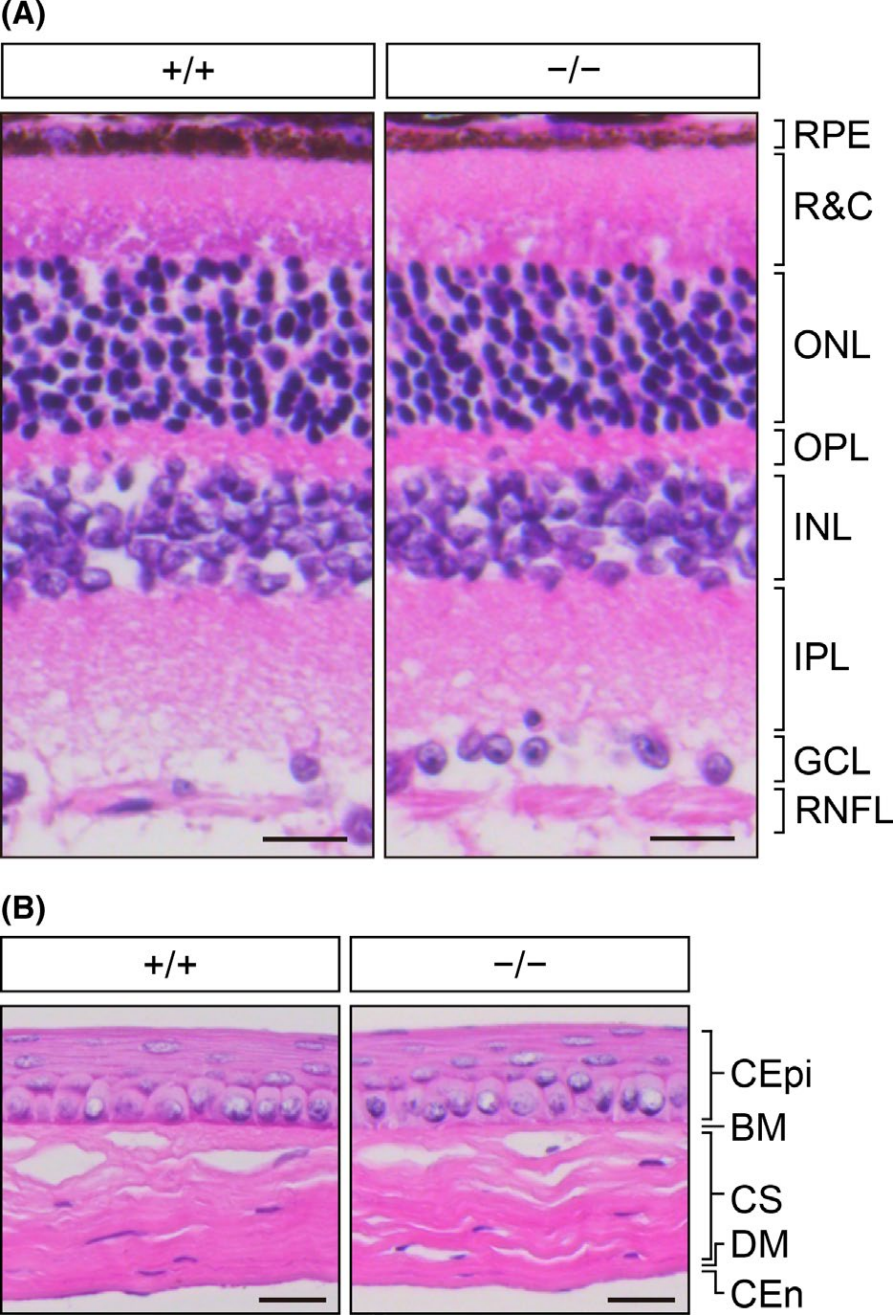
Normal retinal and corneal histology in *Elovl1* ^−/−^ *Tg*(*IVL‐Elovl1*) mice at 16 weeks of age. Representative hematoxylin and eosin staining images of paraffin sections of the retina (A) and the cornea (B) from 16‐week‐old *Elovl1* ^+/+^ *Tg*(*IVL‐Elovl1*) and *Elovl1* ^−/−^ *Tg*(*IVL‐Elovl1*) mice. Scale bars, 20 μm. Abbreviations: (A) GCL, ganglion cell layer; INL, inner nuclear layer; IPL, inner plexiform layer; ONL, outer nuclear layer; OPL, outer plexiform layer; R&C, layer of rods and cones; RNFL, retinal nerve fiber layer; RPE, retinal pigment epithelium. (B) BM, Bowman's membrane; CEn, corneal endothelium; CEpi, corneal epithelium; CS, corneal stroma; DM, Descemet's membrane. +/+, *Elovl1* ^+/+^ *Tg*(*IVL‐Elovl1*) mice; −/−, *Elovl1* ^−/−^ *Tg*(*IVL‐Elovl1*) mice

After completion of the behavioral tests, when the mice were 16 weeks old we performed histological analyses of the retina and cornea, because patients with the *ELOVL1* mutation exhibit retinal pathology such as thinning of the nerve fiber layer in the retina,^17^, ^18^ and aged *Elovl1* ^−/−^ *Tg*(*IVL‐Elovl1*) mice exhibit corneal opacity.^13^ The histological architecture of the retina, including the nerve fiber layer, did not differ detectably between control and *Elovl1* ^−/−^ *Tg*(*IVL‐Elovl1*) mice (Figure 6A). We also found no discernible differences between the corneas of the two groups of mice (Figure 6B). These results suggest the absence of ocular abnormalities in *Elovl1* ^−/−^ *Tg*(*IVL‐Elovl1*) mice.

## 4 DISCUSSION

In their brain tissue, *Elovl1* ^−/−^ *Tg*(*IVL‐Elovl1*) mice had sphingolipids with shortened acyl moieties, with C24 sphingolipids being almost entirely lost (Figure 1). These results are consistent with the substrate specificity of ELOVL1 determined in vitro: ELOVL1 is highly active toward saturated and monounsaturated C20‐ and C22‐CoAs.^12^, ^13^ In addition to the shortening of acyl moieties, the total levels of GalCer and 2‐OH GalCer were significantly reduced in *Elovl1* ^−/−^ *Tg*(*IVL‐Elovl1*) mice (Figure 1C, D). These reductions may be explained by a decrease in the mRNA levels of *Ugt8a*, encoding GalCer synthase (Figure 2A), although how the lack of *Elovl1* affects the expression of *Ugt8a* remains unclear.

*Elovl1* ^−/−^ *Tg*(*IVL‐Elovl1*) mice exhibited mild myelin thinning in the corpus callosum (Figure 3). This abnormality may be associated with the hypomyelination observed in the corpus callosum and other brain regions in patients with the *ELOVL1* mutation. In *Ugt8a*‐knockout mice, in which both GalCer and 2‐OH GalCer and the sulfatides were absent, thinner and split myelin sheaths were observed in the white matter of the spinal cord,^5^, ^6^ suggesting that reductions in GalCer and 2‐OH GalCer underlie the observed myelin thinning in *Elovl1* ^−/−^ *Tg*(*IVL‐Elovl1*) mice. These reductions in GalCer and 2‐OH GalCer in *Elovl1* ^−/−^ *Tg*(*IVL‐Elovl1*) mice may impair the recruitment of PLP1, the major myelin protein essential to myelin function, into lipid rafts rich in GalCer, 2‐OH GalCer, and cholesterol, and this may in turn impair myelin function.^28^, ^29^ Alternatively, myelin membranes with reduced GalCer and 2‐OH GalCer content may have fewer hydrogen bonds between and/or within the myelin layers, which would render them less stable. It is possible that the shortening of acyl moieties in myelin sphingolipids also contributes to the myelin thinning. Giant unilamellar vesicles prepared from microsomal lipids in *Cers2*‐knockout mice have reduced VLC sphingolipids and exhibit morphological changes such as the presence of non‐spherical vesicles with different membrane curvature, small vesicles inside bigger vesicles, and the formation of aggregates.^30^ These changes in the biophysical properties of membranes may affect the formation and/or stability of myelin. In contrast to the deficit in myelin, we did not detect signs of axonal abnormalities. In fact, axon diameters and densities in the corpus callosum were comparable between *Elovl1* ^+/+^ *Tg*(*IVL‐Elovl1*) and *Elovl1* ^−/−^ *Tg*(*IVL‐Elovl1*) mice (Figure 3A, B). Thus, the hypomyelination in patients with the *ELOVL1* mutation may be caused by autonomous cellular defects in oligodendrocytes.

To identify neurological phenotypes related to the neural symptoms seen in patients with the *ELOVL1* mutation, we performed a battery of behavioral tests and identified several abnormalities in *Elovl1* ^−/−^ *Tg*(*IVL‐Elovl1*) mice: reduced body weight, slower travel speed and more paw slips in the balance beam test, decreased acoustic startle response to high‐intensity stimulus, reduced travel distance and fewer transitions between light and dark chambers in the light–dark transition test, and fewer contacts in the social‐interaction test (Figures 4 and 5). Considering that body temperature and food consumption are unaffected in *Elovl1* ^−/−^ *Tg*(*IVL‐Elovl1*) mice (Figure 4B, C), it is possible that their reduced body weight is associated with reduced nutrient absorption from foods. The defects in the balance beam test (slower travel speed and more paw slips on a narrow beam; Figure 4K, L) are presumably related to spastic paraplegia. The balance beam test is a powerful analysis for identifying a subtle defect in motor function even in mice with no apparent spasticity or ambulation difficulty. In fact, we have previously identified similar defects in *Aldh3a2*‐knockout mice using this test.^19^ *Aldh3a2*‐knockout mice serve as a model for Sjögren‐Larsson syndrome,^31^ an autosomal recessive neurocutaneous disorder caused by mutations in the *ALDH3A2* gene and characterized by spastic paraplegia and ichthyosis^32^—symptoms also observed in patients with the *ELOVL1* mutation. ALDH3A2 is a fatty aldehyde metabolizing enzyme, and *Aldh3a2*‐knockout mice exhibit a decrease in 2‐OH GalCer due to inactivation of FA2H, which synthesizes 2‐OH FAs for 2‐OH GalCer production.^19^ These correlations further support our hypothesis that decreases in 2‐OH GalCer are associated with the hypomyelination and spastic paraplegia observed in patients with the *ELOVL1* mutation.

Whereas patients with this mutation suffered from spasticity of the lower legs and became wheelchair‐dependent,^17^, ^18^ the defect in motor function in *Elovl1* ^−/−^ *Tg*(*IVL‐Elovl1*) mice did not involve paraplegia but was instead rather mild (Figure 4K, L). This difference in the extent of motor dysfunction between human and mouse may result from the degree of hypomyelination. Patients with the *ELOVL1* mutation exhibit widespread hypomyelination, as observed in magnetic resonance imaging (MRI),^17^, ^18^ whereas *Elovl1* ^−/−^ *Tg*(*IVL‐Elovl1*) mice exhibit subtle hypomyelination—specifically, myelin thinning in a subpopulation of axons (Figure 3). Thus, in humans, oligodendrocytes may depend more on VLC sphingolipids for myelination formation and maintenance than they do in mice. Another possibility is that the oligodendrocytes themselves may require VLC sphingolipids equally in human and mouse, but that human nerves are more likely to be affected due to their longer axons. Because there is more myelin sheathing along the length of longer axons, human nerves such as the motor nerves that innervate the lower limbs may be more susceptible to the cumulative effect exerted by each defective myelin sheath.

Patients with the *ELOVL1* mutation exhibited abnormalities related to vision, such as decreased visual acuity, astigmatism, photophobia, and thinning of the nerve fiber layer in the retina.^17^, ^18^ *Elovl1* ^−/−^ *Tg*(*IVL‐Elovl1*) mice exhibited a tendency to avoid the central area during the first 45 minutes of the open‐field test and lower activity levels in the light–dark transition test, despite the lack of detectable retinal histologic abnormality (Figures 4, 5, 6). These defects are likely related to differences in luminance, and may be partially relevant to the photophobia observed in one of the two patients. Although aged *Elovl1* ^−/−^ *Tg*(*IVL‐Elovl1*) mice exhibit corneal opacity because of chronic dry‐eye symptoms,^13^ we confirmed that the corneas of these mice exhibited no pathological abnormalities at 16 weeks of age, when the behavioral analyses were completed (Figure 6B). Thus, the deficiency in motor function identified in the balance beam test is likely associated with a defect in the brain rather than in the eye.

The defect in the acoustic startle response test in *Elovl1* ^−/−^ *Tg*(*IVL‐Elovl1*) mice may be related to the high‐frequency hearing deficit in patients with the *ELOVL1* mutation. Mice lacking *St3gal5*, which encodes the enzyme involved in the synthesis of ganglioside GM3 (one of the glycosphingolipids), exhibit hearing deficiency due to degeneration of the organ of Corti in the cochlea.^33^ Thus, VLC gangliosides may be necessary for the detection of high‐frequency sound.

The neurography of the peripheral motor and sensory nerves was normal in patients with the *ELOVL1* mutation.^17^, ^18^ Similarly, *Elovl1* ^−/−^ *Tg*(*IVL‐Elovl1*) mice exhibited normal nociceptive responses in the hot plate test (Figure 4F). These mice also exhibited normal anxiety‐like responses in the elevated plus‐maze test, mostly normal scores except for a slight decrease in the number of contacts in the social‐interaction test, and normal depression‐like responses in the Porsolt forced‐swim test (Figure 5). These results may correlate with the normal development of sociality in patients with the *ELOVL1* mutation.

Combined, the results of this study show that *Elovl1* ^−/−^ *Tg*(*IVL‐Elovl1*) mice exhibit changes in myelin sphingolipid composition and abundance, reduced myelin thickness, and subtle but significant impairment of motor and auditory functions. These results demonstrate the importance of VLC sphingolipids for myelin function, as well as the suitability of *Elovl1* ^−/−^ *Tg*(*IVL‐Elovl1*) mice as a model for the study of human *ELOVL1* deficiency.

## CONFLICT OF INTEREST

The authors declare no conflicts of interest.

## AUTHOR CONTRIBUTIONS

T. Sassa and A. Kihara designed the research and wrote the paper; M. Isokawa, T. Sassa, and S. Hattori performed the experiments and analyzed the data. T. Miyakawa provided technical support for behavioral analyses. All the authors reviewed and approved the final version of the manuscript.
